# Peripheral arterial lesions detected by vascular ultrasound and their association with aortic events in heritable thoracic aortic diseases

**DOI:** 10.1016/j.ijcha.2026.101898

**Published:** 2026-02-27

**Authors:** Maxime Mongault, Amer Hamade, Corina Mirea, Patrick Ohlmann, Philippe Billaud, Anne Lejay, Salima El Chehadeh, Elena-Mihaela Cordeanu, Dominique Stephan

**Affiliations:** a Department of Hypertension and Vascular Diseases, Strasbourg University Hospital, Strasbourg, France; b Department of Vascular Diseases, Mulhouse Hospital, Mulhouse, France; c Division of Cardiology, Strasbourg University Hospital, Strasbourg, France; d Department of Cardiovascular Surgery, Strasbourg University Hospital, Strasbourg, France; e Department of Vascular Surgery and Renal Transplantation, Strasbourg University Hospital, Strasbourg, France; f Department of Medical Genetics, Strasbourg University Hospital, Strasbourg, France

**Keywords:** Heritable thoracic aortic disease, Marfan syndrome, Peripheral arteries, Aneurysm, Vascular ultrasound, Aortic dissection

## Abstract

**Background:**

Peripheral arterial involvement is increasingly recognized in patients with Marfan syndrome and related heritable thoracic aortic diseases (HTAD), yet its prevalence and clinical relevance remain poorly defined. We aimed to assess the prevalence, anatomical distribution, and association with aortic events of primary peripheral arterial lesions (PPAL) in a genetically characterized HTAD cohort.

**Methods:**

We performed a cross-sectional, single-center study including 61 patients with genetically confirmed HTAD who underwent comprehensive pan-arterial vascular ultrasound. PPAL were classified as aneurysm, ectasia, arterial tortuosity, or mega-artery. Associations between PPAL and aortic events (dissection and/or prophylactic surgery) were evaluated.

**Results:**

PPAL were identified in 39 patients (63.9%), totaling 126 lesions. Ectasia was most frequent (39%), followed by tortuosity (33%), aneurysms (25%), and mega-arteries (21%). Supra-aortic trunks were the most commonly affected territory. Peripheral aneurysms were significantly associated with aortic dissection (odds ratio [OR] 3.60, 95% confidence interval [CI] 1.03–12.54, p = 0.050). Ectasia was associated with the composite endpoint of dissection and/or surgery (OR 3.19, p = 0.040). A higher lesion burden (≥2 lesion types) was significantly associated with aortic surgery (OR 3.80, p = 0.032).

**Conclusions:**

PPAL are highly prevalent in HTAD patients and can be systematically identified using vascular ultrasound. Peripheral aneurysms are associated with aortic dissection, suggesting that comprehensive pan-arterial assessment may improve risk stratification.

## 1 Introduction

Marfan syndrome (MFS) is an autosomal dominant connective tissue disorder caused by pathogenic variants in the FBN1 gene, affecting approximately 1 in 5000 individuals worldwide [1], [2], [3]. Cardiovascular manifestations, particularly aortic involvement, remain the leading cause of morbidity and mortality in these patients [2], [4]. Traditionally, clinical attention has focused on aortic root dilation and the prevention of aortic dissection, which are central to the revised Ghent diagnostic criteria [5]. Advances in medical management and prophylactic aortic surgery have significantly improved survival rates in affected individuals [4], [6], [7]. However, emerging evidence suggests that MFS, along with other heritable thoracic aortic diseases (HTAD), represents a systemic vascular disorder, with arterial involvement extending beyond the proximal aorta [3], [8], [9]. This broader vascular phenotype remains incompletely characterized, and its full implications for risk stratification and long-term management are not yet fully understood. Primary peripheral arterial lesions (PPAL) are defined as structural arterial abnormalities originating from the intrinsic connective tissue defect rather than from the extension or propagation of an aortic dissection. These lesions include focal dilatations, such as aneurysms and ectasia, diffuse arterial enlargement (mega-artery), and increased arterial tortuosity [10], [11], [12]. PPAL have been increasingly reported in patients with MFS and related HTADs, with reported prevalence rates ranging from 15% to over 30%, depending on the population and diagnostic criteria used [13], [14], [15], [16], [17], [18]. These lesions may affect multiple vascular territories, including the supra-aortic trunks, as well as the iliac, femoral, popliteal, and visceral arteries, supporting the concept of a systemic arteriopathy driven by the underlying connective tissue defect. However, the clinical significance of PPAL remains poorly defined. In particular, it is unclear whether the extent and distribution of peripheral arterial involvement are associated with a more severe aortic phenotype or with an increased risk of adverse aortic events.

Despite these observations, several key questions remain unanswered. First, the lack of standardized definitions and classification systems for peripheral arterial abnormalities has limited the comparability of studies [19]. Second, the clinical relevance of different types of lesions, such as aneurysms, ectasia, tortuosity, and diffuse arterial enlargement, has not been systematically evaluated. Third, the diagnostic utility of non-invasive imaging modalities, particularly vascular ultrasound, for comprehensive screening of peripheral arterial involvement in routine clinical practice remains uncertain [20], [21], [22]. Finally, the relationship between PPAL and major aortic events, including aortic dissection and the need for prophylactic aortic surgery, has yet to be fully elucidated [12], [14], [17], [18]. The objective of this study was to use a standardized pan-arterial vascular ultrasound protocol to determine the prevalence and anatomical distribution of PPAL in a cohort of genetically characterized patients with HTAD. In addition, we sought to evaluate the association between PPAL and aortic complications, including aortic dissection and the need for prophylactic aortic surgery. By addressing these objectives, this study aims to clarify the clinical significance of peripheral arterial involvement in HTAD, explore its potential role in improving risk stratification, and assess the feasibility of incorporating comprehensive peripheral arterial screening into routine clinical practice.

## 2 Patients and methods

### 2.1 Study design and population

We conducted a cross-sectional, observational, single-center study including consecutive patients with genetically confirmed MFS and related HTAD who underwent a comprehensive pan-arterial ultrasound examination as part of routine clinical care at the Regional Competence Center for Marfan Syndrome and Related Heritable Aortopathies, Strasbourg University Hospital. Patients were included from 2017 onward, corresponding to the initiation of the Regional Competence Center. No additional imaging procedures or interventions were performed for research purposes. All patients had previously provided written informed consent for inclusion in the European Heritable Thoracic Aortic Diseases (HTAD) Registry, a multicenter registry collecting clinical, genetic, and imaging data from patients with rare vascular diseases followed at expert centers across Europe, and authorizing secondary use of data for research. As the present study relied exclusively on existing clinical and imaging data and did not require any additional procedures, it complied with French regulatory requirements for retrospective research using existing health data (MR-004 framework).

### 2.2 Eligibility criteria

This cross-sectional, single-center study included consecutive patients with genetically confirmed HTAD followed at our Regional Competence Center. Eligible patients carried a pathogenic or likely pathogenic variant in a recognized HTAD-associated gene (including FBN1, TGFBR1, TGFBR2, SMAD3, TGFB2, TGFB3, or ACTA2) and had undergone a comprehensive or attempted comprehensive pan-arterial vascular ultrasound examination as part of routine clinical care. Adults (≥18 years) were included; a limited number of minors were also eligible when written consent had been obtained from a parent or legal guardian for participation in the HTAD Registry. Patients with peripheral arterial abnormalities clearly attributable to non-HTAD etiologies (atherosclerotic, inflammatory, traumatic, or iatrogenic) were not considered for analysis. Only patients with sufficient clinical, genetic, and imaging data available for reliable assessment were included.

### 2.3 Data collection

Clinical, genetic, and imaging data were retrospectively extracted from the electronic medical record (DXCare) and institutional databases. Collected variables included demographic characteristics, genetic results, aortic imaging findings (maximal aortic diameter, involved segments, history of aortic dissection and/or prophylactic surgery), detailed results of the pan-arterial ultrasound examination, and information on subsequent clinical management when available. All data were anonymized prior to statistical analysis.

### 2.4 Pan-arterial ultrasound protocol and lesion classification

All ultrasound examinations were performed by experienced vascular imaging physicians using a Philips EPIQ 7 ultrasound system. A multimodal transducer strategy was employed to optimize image quality across vascular territories: L12-5 MHz and eL18-4 MHz linear probes for superficial cervical and peripheral arteries, a S5-2 MHz cardiac sector probe for thoracic aortic assessment when accessible, a C8-5 MHz micro-convex probe for anatomically restricted regions, and a C5-1 MHz abdominal convex probe for visceral and iliac arteries. This approach enabled high-resolution imaging of superficial vessels while ensuring adequate penetration for deeper arterial segments. All examinations combined B-mode imaging for morphological assessment and diameter measurements, color Doppler for flow visualization and lesion characterization, and pulsed-wave Doppler for velocity measurements and hemodynamic evaluation. Examinations followed a standardized acquisition protocol routinely implemented in our center, aiming for the most comprehensive possible evaluation of the supra-aortic trunks, visceral arteries, iliac and femoral and popliteal arteries, and other accessible peripheral vessels. To specifically assess the intrinsic vasculopathy associated with HTAD, only PPAL were considered. Arterial abnormalities related to aortic dissection, including extension of the dissection flap, post-dissection remodeling, or downstream hemodynamic consequences, were systematically excluded. PPAL were classified using operational definitions aligned with published literature and expert consensus. Aneurysm was defined as a focal arterial dilation ≥150% of the reference diameter [19]; ectasia, defined as a mild focal dilation not meeting aneurysm criteria; tortuosity, defined as the presence of ≥2 angulations of the vascular axis within a single plane (including kinking, S-shaped curves, or looping) [10], [11]; and mega-artery, defined as diffuse arterial enlargement.

### 2.5 Study endpoints

The primary endpoint was the association between the presence of at least one PPAL on ultrasound and the occurrence of an aortic event, defined as aortic dissection (any type) or prophylactic aortic surgery. A composite endpoint combining aortic dissection and/or prophylactic aortic surgery was also analyzed. Secondary endpoints included the prevalence, type, and anatomical distribution of PPAL; the relationship between PPAL and aortic characteristics (maximal diameter and involved segments); differences in PPAL profiles between patients with and without aortic events; potential associations between medical therapy and the presence of PPAL; and the feasibility and completeness of the pan-arterial ultrasound examination. Genotype–phenotype analyses were exploratory and inherently limited by the small number of patients carrying non-FBN1 variants.

### 2.6 Statistical analysis

Continuous variables are presented as mean ± standard deviation or median [interquartile range], as appropriate based on their distribution. Categorical variables are reported as counts and percentages. Comparisons between groups (patients with versus without primary peripheral arterial lesions [PPAL]) were performed using Fisher’s exact test for categorical variables and the Mann–Whitney *U* test for continuous variables with non-normal distributions. Associations between the presence of PPAL and aortic events were quantified using odds ratios (ORs) with corresponding 95% confidence intervals (95% CIs). Because the study objective was descriptive and exploratory, and no time-to-event data were available, survival analysis was not performed. Odds ratios were calculated using the standard cross-product method from 2 × 2 contingency tables, with 95% confidence intervals derived using the Woolf log-odds approximation. Fisher’s exact test was used to compute p-values. All tests were two-tailed, and a p-value < 0.05 was considered statistically significant. Statistical analyses were conducted using R software (version 4.4.1).

### 2.7 Ethical considerations

This study was conducted in accordance with the ethical standards of the institutional research committee and with the 1964 Helsinki Declaration and its later amendments. It complied with French regulations governing retrospective research using routinely collected health data (MR-004 framework). All participants had previously provided written informed consent for inclusion in the European Heritable Thoracic Aortic Diseases (HTAD) registry, which authorizes the secondary use of clinical and imaging data for research purposes. Patients were informed of the potential reuse of anonymized data and were excluded if they had expressed opposition.

## 3 Results

### 3.1 Patient characteristics

Sixty-one patients with genetically confirmed heritable thoracic aortic disease (HTAD) were included in this retrospective analysis. Nine patients were excluded from the initial cohort, including five with negative genetic testing results and four with missing echocardiographic data. The cohort was predominantly male (64%), with a mean age of 38.5 ± 17.5 years (Table 1). Most patients carried a pathogenic FBN1 variant (72.1%), while the remaining individuals harbored variants in genes associated with Marfan-related or overlapping connective tissue disorders, including SMAD3, MYLK, TGFBR2, LOX, COL4A1, ACTA2, FLNA, TGFB2, and SLC2A10. The mean Marfan systemic score was 4.6, reflecting frequent multisystem involvement. Ectopia lentis was present in 16.4% of patients, pectus abnormalities in 42.6%, and scoliosis in 44.3%. Conventional cardiovascular risk factors were uncommon: 21.3% of patients reported active or prior smoking, 11.5% had hypertension, and only isolated cases of diabetes or dyslipidemia were observed. Aortic involvement was frequent. Twenty-one patients (34.4%) had undergone prophylactic ascending aortic surgery, most often valve-sparing root replacement (71.4%), at a mean age of 35 years. Sixteen patients (26.2%) had experienced an aortic dissection prior to ultrasound evaluation, predominantly Stanford type A (75%). Mitral valve prolapse was identified in nearly one-third of the cohort. Medical therapy reflected contemporary standards, with 75% of patients receiving β-blockers and 16.4% treated with angiotensin II receptor blockers.

**Table 1:** Baseline demographic, genetic, and clinical characteristics of the study population.

Characteristic	Value N(%)/m ± SD
Age, years	38.5 ± 17.5
Sex, male	40 (65.6%)
BMI, kg/m^2^	22.5 ± 4.4
Arm span/height ratio	1.04 ± 0.04
Systemic score	4.6 ± 3.35
Ectopia lentis	10 (16.4%)
Pectus deformity	26 (42.6%)
Scoliosis	27 (44.3%)
Gene involved	
FBN1	44 (72.1%)
SMAD3	4 (6.6%)
MYLK	4 (6.6%)
TGFBR2	2 (3.3%)
LOX	2 (3.3%)
COL4A1	1 (1.6%)
ACTA2	1 (1.6%)
FLNA	1 (1.6%)
TGFβ2	1 (1.6%)
SLC2A10	1 (1.6%)
CV risk factor	
Active smoking	13 (21.3%)
Arterial hypertension	7 (11.5%)
Diabetes mellitus	1 (1.6%)
Dyslipidemia	2 (3.3%)
CV manifestations and treatment	
History of aortic dissection	16 (26.2%)
Type A	12 (75% of dissections)
Type B	4 (25% of dissections)
Mean age at dissection	39.1 ± 12.1 years
Ascending aortic surgery	21 (34.4%)
Valve-sparing (David)	15 (71.4%)
Bentall procedure	3 (14.3%)
Mitral valve prolapse	19 (31.1%)
Therapy β-blocker	46 (75.4%)
ARB	10 (16.4%)

Abbreviations: ARB: angiotensin II receptor blocker; BMI: body mass index; CV: cardiovascular; m: mean; N:number; SD: standard deviation.

## 4 Prevalence and distribution of peripheral arterial lesions

**Fig. 1:**
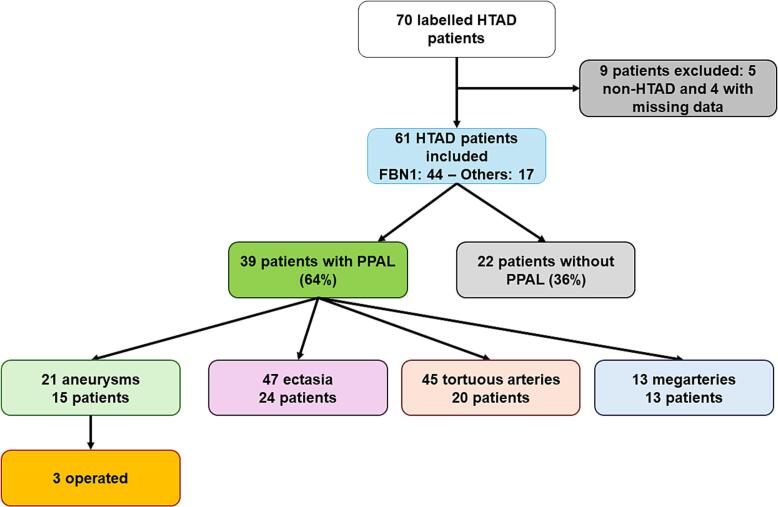
Flowchart of the study cohort and distribution of peripheral arterial abnormalities. Flowchart showing the 61 patients with Marfan syndrome or related heritable thoracic aortic diseases included in the study, the 39 patients (63.9%) with at least one primary peripheral arterial abnormality, and the distribution of the four lesion types identified on pan-arterial ultrasound (aneurysms, ectasias, tortuous arteries, and mega-arteries).

PPAL were identified in 39 patients (63.9%) (Fig. 1). A total of 126 individual peripheral arterial abnormalities were recorded (Table 2). Ectasia was the most frequent lesion type, observed in 24 patients (39%), accounting for 47 lesions. These were predominantly located in the supra-aortic vessels, most commonly the internal carotid (n = 11), followed by subclavian/axillary (n = 7), and vertebral arteries (n = 6). Additional ectasias were identified in the iliac (n = 11), femoral (n = 6), and popliteal arteries (n = 4). Isolated ectasias were also observed in other vascular territories, including one coronary abnormality reported on prior imaging and one mesenteric lesion detected by ultrasound. Arterial tortuosity was identified in 20 patients (33%), corresponding to 45 lesions, with a marked predominance in the cervical arteries (76% of cases). Eight lesions involved the iliac system, with isolated cases affecting the splenic and femoral arteries. Tortuosity was more frequently observed in patients with FBN1 variants, suggesting a possible genotype–phenotype association, although this finding should be interpreted cautiously given the limited number of non-FBN1 genotypes. Peripheral aneurysms were detected in 15 patients (24.6%), representing 21 lesions. Nearly half were located in the supra-aortic trunks, particularly the vertebral and internal carotid arteries, while approximately one-third involved the iliac arteries. Single aneurysms were identified in the celiac trunk, splenic, renal, and common femoral arteries. Three patients underwent peripheral aneurysm surgery: one patient with bilateral axillary artery aneurysms (right 110 mm, left 40 mm), one with a right common iliac artery aneurysm (37 mm), and one with a right subclavian artery aneurysm (30 mm). Diffuse arterial enlargement consistent with mega-artery was observed in 13 patients (21%), predominantly affecting the iliofemoral axis. Arterial diameter measurements for aneurysms and ectasias are presented in Supplementary Table 1.

**Table 2:** Distribution of primary peripheral arterial lesions.

Type of abnormality	Number of lesions	Anatomical distribution
Aneurysm	21	Supra-aortic trunks (10) Iliac (7) Celiac trunk (1) Splenic (1) Renal (1) Common femoral (1) - Vertebral (4) - Internal carotid (3) - Subclavian/Axillary (3)
Ectasia	47	Supra-aortic trunks (24) − Internal carotid (11) − Subclavian/Axillary (7) − Vertebral (6) Iliac (11) Femoral (6) Popliteal (4) Left anterior descending coronary (1) Superior mesenteric (1)
Tortuosity	45	Cervical (34) Iliac (8) Splenic (1) Femoral artery (1)
Mega-artery	13	Iliac (13)

### 4.1 Factors associated with PPAL

Patients with PPAL were significantly older than those without PPAL (43.9 ± 16.8 vs. 32.8 ± 16.6 years, p = 0.015). In contrast, sex, body mass index, height-to-arm-span ratio, cardiovascular risk factors, and Marfan systemic score were not associated with the presence of PPAL (Supplementary Table 2). No clear differences in genotype distribution were observed between patients with and without PPAL, although statistical power was limited for non-FBN1 variants.

### 4.2 Association between PPAL and aortic events

**Fig. 2:**
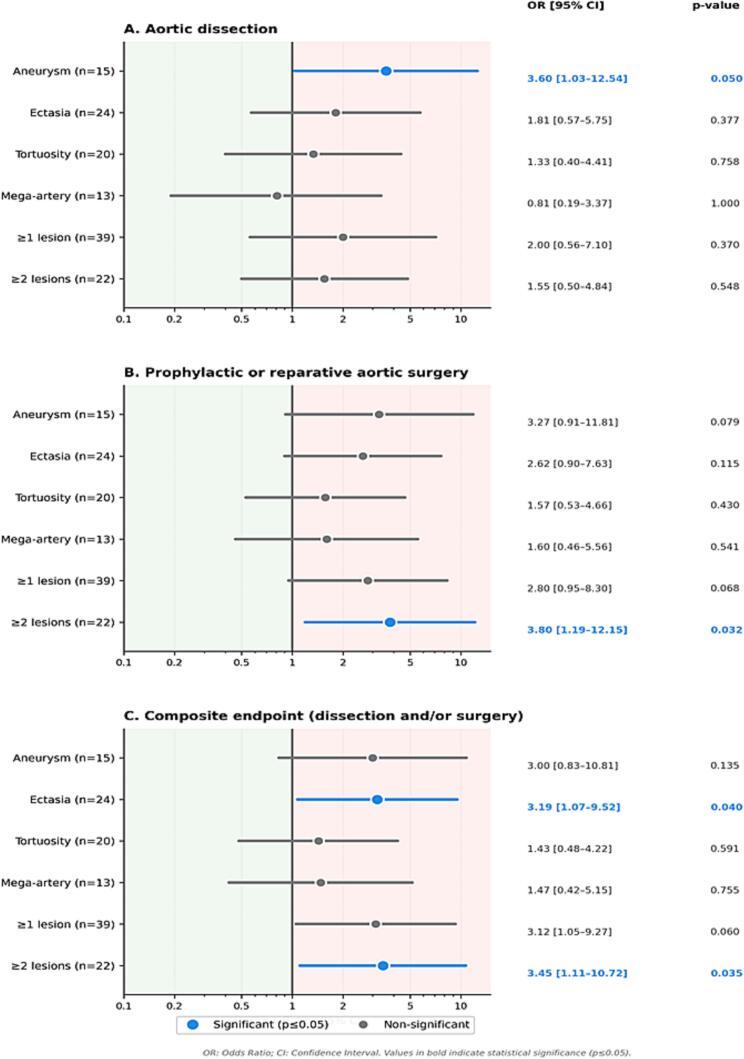
Association between primary peripheral arterial lesions and aortic events. Forest plots showing odds ratios (OR) with 95% confidence intervals (CI) for the association between primary peripheral arterial lesion types and aortic outcomes: (A) aortic dissection, (B) prophylactic or reparative aortic surgery, and (C) composite endpoint (aortic dissection and/or surgery). Individual lesion types (aneurysm, ectasia, tortuosity, mega-artery) and lesion burden (≥1 lesion, ≥2 lesion types) are shown. Blue circles indicate statistically significant associations (p ≤ 0.05); grey circles indicate non-significant associations. The vertical line represents an OR of 1 (no association). OR and 95% CI were calculated using the Woolf log-odds approximation; p-values were derived from Fisher's exact test. (For interpretation of the references to colour in this figure legend, the reader is referred to the web version of this article.)

Among the different lesion types, peripheral aneurysms showed the strongest association with adverse aortic outcomes (Fig. 2, Supplementary Table 3). Patients with peripheral aneurysms were significantly more likely to have experienced an aortic dissection (odds ratio [OR] 3.60, 95% CI 1.03–12.54, p = 0.050). No significant association was observed between aneurysms and prior prophylactic aortic surgery. Ectasias were not independently associated with aortic dissection or prophylactic surgery. However, they were significantly associated with the composite endpoint combining aortic dissection and/or prophylactic surgery (OR 3.19, p = 0.040), suggesting a potential contribution to global aortic risk. Arterial tortuosity and mega-artery were not significantly associated with aortic dissection or surgical intervention. The presence of at least one PPAL was not independently associated with aortic dissection or prophylactic surgery; however, a trend toward an association was observed for the composite endpoint (OR 3.12, 95% CI 1.05–9.27, p = 0.060). By contrast, a higher peripheral lesion burden (≥2 lesion types) was significantly associated with prophylactic or reparative aortic surgery (OR 3.80, p = 0.032) and with the composite endpoint (OR 3.45, p = 0.035). Patients with aneurysms and those with arterial tortuosity tended to experience aortic events at a younger age, although these differences did not reach statistical significance.

## 5 Medical therapy and feasibility of ultrasound assessment

No association was observed between medical therapy and the presence of PPAL. The prevalence of PPAL did not differ between patients receiving β-blockers and those not treated with β-blockers (p = 0.763), nor between patients treated or not treated with angiotensin II receptor blockers (p = 0.305). Overall, comprehensive or near-comprehensive pan-arterial ultrasound assessment was feasible in the majority of patients, enabling systematic evaluation of multiple peripheral vascular territories in routine clinical practice.

## 6 Discussion

In this study of genetically confirmed HTAD, PPAL were highly prevalent, affecting nearly two-thirds of patients. Using standardized operational definitions aligned with published literature and a comprehensive pan-arterial vascular ultrasound approach, we identified widespread peripheral arterial involvement, supporting the concept of HTAD as a systemic arteriopathy extending beyond the thoracic aorta [3], [23]. Among the various lesion types, peripheral aneurysms showed the strongest association with adverse aortic outcomes, suggesting a potential role in risk stratification. The prevalence of PPAL in our cohort (63.9%) exceeds that reported in most previous studies, likely reflecting our systematic screening strategy and the use of comprehensive vascular ultrasound rather than selective or symptom-driven imaging. Prior studies have reported peripheral or branch artery aneurysms in 20–31% of patients with MFS, with consistent associations with adverse outcomes [13], [14], [15], [16], [17], [18]. Yetman et al. reported distal aortic and peripheral aneurysms in 31% of patients with Marfan syndrome, with an association between distal disease and prior aortic root replacement (OR 1.75, 95% CI 1.03–2.98) (Table 3) [18]. Lopez-Sainz et al. demonstrated a higher risk of aortic surgery in patients with branch artery aneurysms (HR 3.4, 95% CI 1.1–10.3) [14]. Pellenc et al. reported a strong association between peripheral arterial aneurysms requiring surgery and aortic dissection in a large FBN1 registry cohort (OR 13.18, 95% CI 5.25–33.05), although the overall prevalence of surgical peripheral aneurysms was low (1.2%), likely reflecting under-detection due to non-systematic imaging [15]. Sénémaud et al. identified PPAL independently associated with aortic dissection (OR 3.90, 95% CI 1.25–12.07) [17]. Taken together, these selected studies contextualize our findings, indicating that peripheral arterial involvement in HTAD is common yet frequently underrecognized, and that the association observed between peripheral aneurysms and aortic dissection in our cohort (OR 3.60, 95% CI 1.03–12.54) is consistent with prior reports. A key observation of this study is the differential prognostic significance of PPAL subtypes. Peripheral aneurysms were associated with a more than threefold increased odds of aortic dissection, supporting the hypothesis that aneurysmal peripheral involvement reflects a more severe connective tissue phenotype and increased vulnerability of the aortic wall [14], [17], [18]. In contrast, ectasia, arterial tortuosity, and diffuse arterial enlargement were not independently associated with dissection, although ectasia was associated with the composite endpoint of dissection and/or prophylactic surgery. Beyond individual lesion subtypes, our results indicate that a higher peripheral arterial lesion burden is clinically relevant: patients presenting with two or more distinct PPAL types had significantly increased odds of undergoing prophylactic or reparative aortic surgery, as well as of experiencing the composite aortic endpoint. These findings suggest that while all PPAL reflect the underlying vasculopathy, peripheral aneurysms may represent a particularly high-risk manifestation. The need for vascular intervention in several asymptomatic peripheral aneurysms, including cases that were already of surgical size at the time of detection, emphasizes the prognostic relevance of these lesions and the potential benefit of systematic screening strategies [24], [25]. The anatomical distribution of PPAL reinforces the systemic nature of vascular involvement in HTAD. The supra-aortic trunks were the most frequently affected vessels, particularly by ectasia and tortuosity, with predominant involvement of the internal carotid, vertebral, and subclavian arteries. This pattern is consistent with previous reports and may reflect specific biomechanical or developmental susceptibilities of cervical arteries [10], [12], [26]. The iliofemoral axis was the second most frequently involved territory and the predominant site of diffuse arterial enlargement, as well as a common location for aneurysms and ectasia [16], [18]. These findings highlight the limitations of surveillance strategies focused exclusively on the thoracic and abdominal aorta [27], [28]. Patients with PPAL were significantly older than those without, suggesting a time-dependent accumulation or progression of peripheral arterial abnormalities, similar to what is observed for aortic disease in Marfan syndrome [4], [29]. This observation supports the potential value of longitudinal vascular surveillance. We did not observe an association between PPAL and medical therapy, including β-blockers or angiotensin II receptor blockers [6], [7]; however, the cross-sectional design and limited sample size preclude conclusions regarding treatment effects on peripheral arterial remodeling.

**Table 3:** Summary of selected studies reporting peripheral arterial aneurysms (PAA) and associated aortic events in heritable thoracic aortic diseases.

Study	Total n	Genotype	PAA n (%)	PAA requiring surgery § n (%)	Outcome n (%)	HR/OR [95% CI]
Yetman, 2011	140	MFS (clinical diagnosis; FBN1 predominant)	44 (31.4%)	NA	Aortic surgery	OR 1.75 [1.03–2.98]
Lopez-Sainz, 2021	187	MFS/FBN1 predominant	50 (26.7%)	5 (2.7%)	Aortic surgery	HR 3.4 [1.1–10.3]
Pellenc, 2022	1575	100% FBN1	NA	19 (1.2%)	Aortic dissection	OR 13.18 [5.25–33.05] †
Sénémaud, 2023	138	100% FBN1	28 (20.3%)	4 (2.9%)	Aortic dissection	OR 3.90 [1.25–12.07]
Present study	61	HTAD/FBN1 predominant	15 (24.6%)	3 (4.9%)	Aortic dissection	OR 3.60 [1.03–12.54]

Abbreviations: HR, hazard ratio; HTAD, heritable thoracic aortic disease; MFS, Marfan syndrome; n, number; OR, odds ratio; PAA, peripheral arterial aneurysm; CI, confidence interval. § Results expressed as number (%) of patients. † Odds ratios and 95% confidence intervals for Pellenc et al. were calculated by the authors from data reported in the original publication.

From a clinical perspective, our study demonstrates the feasibility of comprehensive peripheral arterial assessment using vascular ultrasound in routine practice [20], [21], [22]. This non-invasive and radiation-free technique allows systematic evaluation of multiple vascular territories and can be readily integrated into existing surveillance protocols without additional cross-sectional imaging [27], [28], [30]. The use of standardized consensus-based definitions enhances reproducibility and supports broader implementation across centers [19], [31].

Several limitations should be acknowledged. The cross-sectional, single-center design and modest sample size limit generalizability. The predominance of FBN1 variants restricts genotype–phenotype analyses for rarer HTAD-associated genes [12]. The cross-sectional nature of the study precludes assessment of PPAL progression and their predictive value for future aortic events. In addition, although standardized lesion definitions were applied, external validation in independent cohorts is required. Furthermore, although lesions were compared with CT angiography when available, systematic multimodal confirmation was not performed for all patients and all vascular territories. This should be acknowledged as a limitation, particularly for visceral and deeply located vessels where ultrasound accuracy may be reduced. Prospective multicenter studies are needed to confirm these findings and clarify the role of PPAL in HTAD risk stratification.

## 7 Conclusion

Primary peripheral arterial lesions are highly prevalent in patients with genetically confirmed heritable thoracic aortic diseases and can be systematically identified using comprehensive vascular ultrasound. Peripheral aneurysms are significantly associated with aortic dissection, suggesting that they may serve as markers of a more severe vascular phenotype. These findings support the integration of pan-arterial vascular assessment into routine surveillance strategies for patients with Marfan syndrome and related disorders.

Declaration of Generative AI

Generative AI tools (ChatGPT and Claude) were used solely to assist with language editing and stylistic refinement. All content was reviewed, verified, and approved by the authors, who take full responsibility for the final manuscript.

Statement of authorship: This author takes responsibility for all aspects of the reliability and freedom from bias of the data presented and their discussed interpretation

## CRediT authorship contribution statement

**Maxime Mongault:** Project administration, Methodology, Investigation, Formal analysis, Data curation. **Amer Hamade:** Validation, Methodology, Investigation, Data curation. **Corina Mirea:** Validation, Methodology, Investigation, Data curation. **Patrick Ohlmann:** Visualization, Validation, Resources, Project administration, Conceptualization. **Philippe Billaud:** Validation, Supervision, Formal analysis, Data curation, Conceptualization. **Anne Lejay:** Validation, Supervision, Methodology, Formal analysis, Data curation, Conceptualization. **Salima El Chehadeh:** Supervision, Methodology, Investigation, Formal analysis, Data curation, Conceptualization. **Elena-Mihaela Cordeanu:** Writing – original draft, Visualization, Validation, Supervision, Methodology, Investigation, Formal analysis, Data curation, Conceptualization. **Dominique Stephan:** Writing – review & editing, Writing – original draft, Validation, Supervision, Methodology, Formal analysis, Data curation, Conceptualization.

## Funding

This research did not receive any specific grant from funding agencies in the public, commercial, or not-for-profit sectors.

## Declaration of competing interest

The authors declare that they have no known competing financial interests or personal relationships that could have appeared to influence the work reported in this paper.
