# Supplementary material for: Peripheral arterial lesions detected by vascular ultrasound and their association with aortic events in heritable thoracic aortic diseases
Source: Int J Cardiol Heart Vasc. 2026 Feb 27;63:101898. doi: 10.1016/j.ijcha.2026.101898 (PMC12966744; doi:10.1016/j.ijcha.2026.101898)
Supplement: Supplementary Data 1 [file mmc1.docx]

## Supplementary Table 2. Baseline demographic and clinical characteristics of patients with HTAD according to the presence of PPAL

| **Characteristic** | **Patients with PPAL**  n(%)/m±SD | **Patients without PPAL**  n(%)/m±SD | **p value** |
| --- | --- | --- | --- |
| **n** | **39 (63.9)** | **22 (36.1)** |  |
| Male sex, n (%) | 24 (62) | 16 (72.7) | 0.454 |
| Age, years | 43.9 ± 16.8 | 32.8 ± 16.6 | **0.015** |
| Arm span/height ratio | 1.037 ± 0.041 | 1.036 ± 0.032 | 0.959 |
| BMI, kg/m² | 22.7 ± 4.5 | 22.3 ± 4.2 | 0.720 |
| Systemic score | 5.56 ± 3.39 | 5 ± 2.71 | 0.531 |
| Ectopia lentis, n (%) | 6 (15.4) | 4 (18.2) | 1.000 |
| Pectus deformity, n (%) | 19 (48.7) | 7 (31.8) | 0.282 |
| Scoliosis, n (%) | 20 (51.3) | 7 (31.8) | 0.184 |
| Hypertension | 6 (15.4) | 1 (4.5) | 0.405 |
| Diabetes mellitus | 1 (2.6) | 0 (0) | 1.000 |
| Dyslipidemia | 1 (2.6) | 1 (4.5) | 1.000 |
| Active smoking | 9 (23.1) | 4 (18.2) | 0.753 |
| Beta-blocker therapy | 30 (76.9) | 16 (72.7) | 0.763 |
| ARB therapy | 8 (20.5) | 2 (9.1) | 0.305 |

Abbreviations: ARB: angiotensin II receptor blocker; BMI: body mass index; HTAD: heritable thoracic aortic disease; m: mean; n: number; PPAL: primary peripheral arterial lesions; SD: standard deviation.
