# Supplementary material for: Peripheral arterial lesions detected by vascular ultrasound and their association with aortic events in heritable thoracic aortic diseases
Source: Int J Cardiol Heart Vasc. 2026 Feb 27;63:101898. doi: 10.1016/j.ijcha.2026.101898 (PMC12966744; doi:10.1016/j.ijcha.2026.101898)
Supplement: Supplementary Data 2 [file mmc2.docx]

**Supplementary Table 3.** Association between primary peripheral arterial lesions and aortic events

| **PPAL** | **Aortic dissection** | **Prophylactic or reparative aortic surgery** | **Prophylactic aortic surgery only** | **Composite endpoint *(dissection and/or aortic surgery)*** |
| --- | --- | --- | --- | --- |
| **Individual lesions** | | | | |
| **Aneurysm**  **(n=15)** | 7/15 vs 9/46  **OR 3.60 [1.03–12.54], p = 0.050** | 11/15 vs 21/46  OR 3.27 [0.91–11.81], p = 0.079 | 6/15 vs 15/46  OR 1.38 [0.41–4.59], p = 0.755 | 11/15 vs 22/46  OR 3.00 [0.83–10.81], p = 0.135 |
| **Ectasia**  **(n=24)** | 8/24 vs 8/37  OR 1.81 [0.57–5.75], p = 0.377 | 16/24 vs 16/37  OR 2.62 [0.90–7.63], p = 0.115 | 11/24 vs 10/37  OR 2.28 [0.77–6.76], p = 0.171 | 17/24 vs 16/37  **OR 3.19 [1.07–9.52], p = 0.040** |
| **Tortuosity**  **(n=20)** | 6/20 vs 10/41  OR 1.33 [0.40–4.41], p = 0.758 | 12/20 vs 20/41  OR 1.57 [0.53–4.66]; p = 0.430 | 8/20 vs 13/41  OR 1.44 [0.47–4.43], p = 0.574 | 12/20 vs 21/41  OR 1.43 [0.48–4.22], p = 0.591 |
| **Mega-artery**  **(n=13)** | 3/13 vs 13/48  OR 0.81 [0.19–3.37], p = 1.000 | 8/13 vs 24/48  OR 1.60 [0.46–5.56], p = 0.541 | 6/13 vs 15/48  OR 1.89 [0.54–6.62], p = 0.341 | 8/13 vs 25/48  OR 1.47 [0.42–5.15], p = 0.755 |
| **Lesion burden** | | | | |
| **At least one lesion**  **(n=39)** | 12/39 vs 4/22  OR 2.00 [0.56–7.10], p = 0.370 | 24/39 vs 8/22  OR 2.80 [0.95–8.30], p = 0.068 | 16/39 vs 5/22  OR 2.37 [0.72–7.78], p = 0.173 | 25/39 vs 8/22  OR 3.12 [1.05–9.27], p = 0.060 |
| **≥2 lesions**  **(n=22)** | 7/22 vs 9/39  OR 1.55 [0.50–4.84], p=0.548 | 16/22 vs 16/39  **OR 3.80 [1.19–12.15], p=0.032** | 11/22 vs 10/39  OR 2.90 [0.96–8.79], p=0.091 | 16/22 vs 17/39  **OR 3.45 [1.11–10.72], p=0.035** |

N: number of patients; OR : odd ratio. PPAL: primary peripheral arterial lesions; Values are number of events / total (%) in patients with the lesion vs without the lesion, followed by odds ratio (OR) with 95% confidence interval estimated using the Woolf log-odds approximation and Fisher exact p-value.
