# Supplementary material for: Peripheral arterial lesions detected by vascular ultrasound and their association with aortic events in heritable thoracic aortic diseases
Source: Int J Cardiol Heart Vasc. 2026 Feb 27;63:101898. doi: 10.1016/j.ijcha.2026.101898 (PMC12966744; doi:10.1016/j.ijcha.2026.101898)
Supplement: Supplementary Data 3 [file mmc3.docx]

**Supplementary Table 1.** Arterial diameter measurements for aneurysms and ectasias.

| **Vascular territory** | **n** | **Median [IQR] (mm)** | **Min–Max (mm)** |
| --- | --- | --- | --- |
| ***Aneurysms (n = 21)*** | | | |
| **Supra-aortic trunks** | | | |
| Vertebral artery | 4 | 7.5 [6.4–12] | 6.4–12 |
| Internal carotid artery | 3 | 10.3 [10–15.9] | 10–15.9 |
| Subclavian/Axillary artery | 3 | 40 [29–110] | 29–110 |
| Iliac arteries | 7 | 20 [19–22] | 17–37 |
| Celiac trunk | 1 | / | / |
| Splenic artery | 1 | 13 | / |
| Renal artery | 1 | 12.4 | / |
| Common femoral artery | 1 | 18.4 | / |
| ***Ectasias (n = 47)*** | | | |
| **Supra-aortic trunks** | | | |
| Internal carotid artery | 11 | 11 [10–12] | 8–12 |
| Subclavian/Axillary artery | 7 | 14 [12–17] | 10.4–18 |
| Vertebral artery | 6 | 5.75 [5.5–6] | 5–7 |
| Iliac arteries | 11 | 13.4 [12–14] | 12–15 |
| Femoral arteries | 6 | 12 [11.7–12.4] | 10.1–13.9 |
| Popliteal arteries | 4 | 9.5 [9–14] | 9–14 |
| Coronary artery (LAD) | 1 | / | / |
| Superior mesenteric artery | 1 | 11.5 | / |

Abbreviations: IQR, interquartile range; LAD, left anterior descending; NA, not available;

Note: For territories with a single lesion, only the measured diameter is reported (range not applicable). Diameter data were not available for one celiac trunk aneurysm and one coronary ectasia identified on prior imaging.
