# Supplementary material for: Peripheral arterial lesions detected by vascular ultrasound and their association with aortic events in heritable thoracic aortic diseases
Source: Int J Cardiol Heart Vasc. 2026 Feb 27;63:101898. doi: 10.1016/j.ijcha.2026.101898 (PMC12966744; doi:10.1016/j.ijcha.2026.101898)
Supplement: Supplementary Data 4 [file mmc4.docx]

**Supplementary Table 1.** Arterial diameter measurements for aneurysms and ectasias.

| **Vascular territory** | **n** | **Mean ± SD (mm)** | **Median [IQR] (mm)** | **Min–Max (mm)** |
| --- | --- | --- | --- | --- |
| ***Aneurysms (n = 21)*** | | | | |
| **Supra-aortic trunks** | | | | |
| Vertebral artery | 4 | 8.35 ± 2.48 | 7.5 [6.4–12] | 6.4–12 |
| Internal carotid artery | 3 | 12.07 ± 3.27 | 10.3 [10–15.9] | 10–15.9 |
| Subclavian/Axillary artery | 3 | 59.67 ± 43.93 | 40 [29–110] | 29–110 |
| Iliac arteries | 7 | 22.14 ± 6.72 | 20 [19–22] | 17–37 |
| Celiac trunk | 1 | NA | / | / |
| Splenic artery | 1 | 13 | / | / |
| Renal artery | 1 | 12.4 | / | / |
| Common femoral artery | 1 | 18.4 | / | / |
| ***Ectasias (n = 47)*** | | | | |
| **Supra-aortic trunks** | | | | |
| Internal carotid artery | 11 | 10.61 ± 1.30 | 11 [10–12] | 8–12 |
| Subclavian/Axillary artery | 7 | 14.49 ± 2.90 | 14 [12–17] | 10.4–18 |
| Vertebral artery | 6 | 5.83 ± 0.54 | 5.75 [5.5–6] | 5–7 |
| Iliac arteries | 11 | 13.27 ± 1.08 | 13.4 [12–14] | 12–15 |
| Femoral arteries | 6 | 12.02 ± 1.22 | 12 [11.7–12.4] | 10.1–13.9 |
| Popliteal arteries | 4 | 10.50 ± 2.35 | 9.5 [9–14] | 9–14 |
| Coronary artery (LAD) | 1 | NA | / | / |
| Superior mesenteric artery | 1 | 11.5 | / | / |

*Abbreviations: IQR, interquartile range; LAD, left anterior descending; NA, not available; SD, standard deviation.*

*Note: For territories with a single lesion, only the measured diameter is reported (median and range not applicable). Diameter data were not available for one celiac trunk aneurysm and one coronary ectasia identified on prior imaging.*
